# Development and Application of an Atmospheric Pollutant Monitoring System Based on LoRa—Part I: Design and Reliability Tests

**DOI:** 10.3390/s18113891

**Published:** 2018-11-12

**Authors:** Yushuang Ma, Long Zhao, Rongjin Yang, Xiuhong Li, Qiao Song, Zhenwei Song, Yi Zhang

**Affiliations:** 1 College of Global Change and Earth System Science, Beijing Normal University, No.19, Xinjiekou Wai Street, Haidian District, Beijing 100875, China; mays@mail.bnu.edu.cn (Y.M.); zhaolong_ak@163.com (L.Z.); zgsongqiao@163.com (Q.S.); szwzsx@126.com (Z.S.); 201721490029@mail.bnu.edu.cn (Y.Z.); 2 Chinese Research Academy of Environmental Sciences, No.8, Da Yang Fang, An Wai, Chao Yang, Beijing 100012, China; yangrj@craes.org.cn; 3 State Key Laboratory of Remote Sensing Science, Jointly Sponsored by Beijing Normal University and Institute of Remote Sensing and Digital Earth of Chinese Academy of Sciences Beijing Normal University, Beijing 100101, China

**Keywords:** atmospheric, on-line monitoring, LoRa, embedded system

## Abstract

At present, as growing importance continues to be attached to atmospheric environmental problems, the demand for real-time monitoring of these problems is constantly increasing. This article describes the development and application of an embedded system for monitoring of atmospheric pollutant concentrations based on LoRa (Long Range) wireless communication technology, which is widely used in the Internet of Things (IoT). The proposed system is realized using a combination of software and hardware and is designed using the concept of modularization. Separation of each function into independent modules allows the system to be developed more quickly and to be applied more stably. In addition, by combining the requirements of the remote atmospheric pollutant concentration monitoring platform with the specific requirements for the intended application environment, the system demonstrates its significance for practical applications. In addition, the actual application data also verifies the sound application prospects of the proposed system.

## 1. Introduction

**Figure 1:**
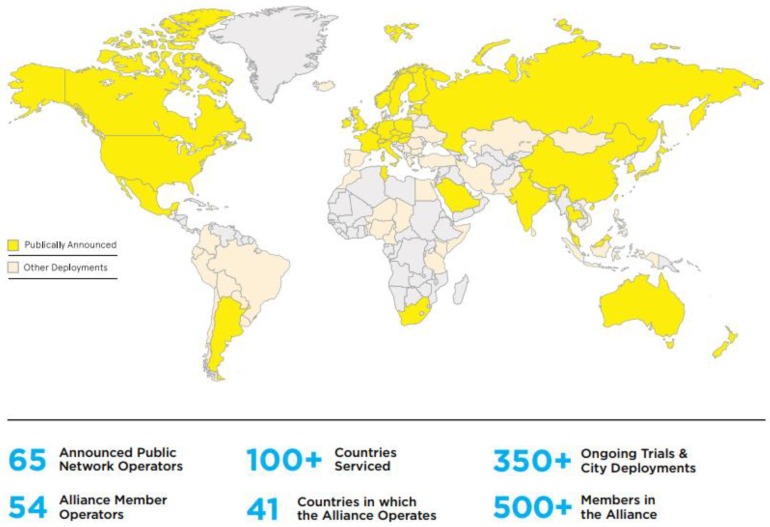
Global distribution of long range (LoRa) usage (which was made by the LoRa alliance and can be found at https://lora-alliance.org/). The dark yellow parts indicate that the application of LoRa technology has been publicly announced, the light-yellow parts indicate that the LoRa technology has a small number of applications, and the light grey parts indicate that the LoRa technology has not yet been applied.

Atmospheric pollution is an important issue in China because the severe effects of this pollution are detrimental to the life and health of the people [1]. The main sources of atmospheric pollution include organic compounds, carbon compounds, and particulate matter (PM) produced by human activities, including nitrogen dioxide, carbon monoxide and PM2.5, which can all seriously affect human health [2]. To provide better solutions to the problem of atmospheric pollution, it is essential to identify the source of each pollution discharge accurately and rapidly. However, at present, atmospheric monitoring in China is still reliant on large-scale national monitoring stations, which can provide high-precision monitoring data but are not arranged in large-area and high-density monitoring sites because of cost constraints. As a result, we cannot acquire the monitoring data with sufficiently high spatial precision to locate the discharge sources. Therefore, it is essential that monitoring sites that can acquire data with high space-time accuracy are developed. With the development of the Internet of Things (IoT) [3], the number of IoT devices in general use is continuing to grow rapidly. Application of the IoT to monitor the air quality can provide sufficient data to allow the pollution sources to be located precisely. With characteristics that include on-line monitoring, target tracking, remote maintenance, and an on-line upgrade capability, the IoT can solve the problems of high-space-time-precision air monitoring efficiently by selecting the most appropriate networking mode to realize interconnection and communication for air monitoring equipment. The emerging LoRa (Long Range) [4] is a type of low-power wireless area network (WAN) communication technology that offers several advantages when applied to the IoT. As shown in Figure 1, LoRa applications are becoming more widespread. Using a star network structure, LoRa achieves ultra-low current consumption and provides long-distance transmission via a spread-spectrum modulation design and a reduced communication modulation frequency, and it is resistant to external interference and multipath fading because it uses frequency-hopping spread spectrum technology. These IoT-compatible features make it suitable for device applications that require low power consumption and small data volumes (less than 50 bytes for a single packet). To date, many countries have started to develop applications for the IoT, and some of the required infrastructure, such as LoRa base stations, has been deployed extensively [5].

In addition, a great deal of research is being conducted into the application of LoRa to IoT. For example, in China, Wang et al. realized a power meter reading module through use of LoRa [6], Sun et al. applied LoRa technology to power a communications network [7], and the study of long-distance indoor location functions have also been performed based on LoRa’s localizable features [8]. Overseas, Pasolini et al. studied the application of LoRa to smart city projects [9], while Cerchecci et al. conducted research into the use of LoRa for urban domestic garbage collection management [10]. These numerous research areas demonstrate the current development prospects of LoRa. There are also some research results on atmospheric environmental monitoring methods. Olalekan A.M. et al. proposed using networks of low-cost air quality sensors to quantify air quality in urban settings [11]. The study introduced the collection of pollutant concentrations by using general packet radio service (GPRS) network communication, and the measurement results of the electrochemical sensor are analyzed in detail. L. Capezzuto et al. also proposed an interesting monitoring method through mobile phones to provide a way for the citizen to easily obtain his own sensor node and quickly start participating in the air monitoring [12]. M.I. Mead et al. provided evidence for the performance of electrochemical sensors at the parts-per-billion level, and then they outlined results obtained from deployments of networks of sensor nodes in an autonomous, high-density, static network in the wider Cambridge (UK) area [13]. Therefore, it can be seen from the above studies that monitoring of the atmospheric environment is a research hotspot and there is also a large market demand.

Based on the above research, this article introduces a high-time-space-precision atmospheric environmental monitoring system based on LoRa (which is hereafter referred to as “the system”). The system was designed and implemented based on the requirements of field environment monitoring applications. The system is modular in terms of both hardware and software to enable it to meet later expansion requirements. Furthermore, the system was designed with full consideration of aspects such as equipment power consumption, reliability, hardware volume, and actual cost. The actual application also demonstrated that the system has strong market prospects. The design, implementation and application of the proposed system are described in detail below.

## 2. System Structure

**Figure 2:**
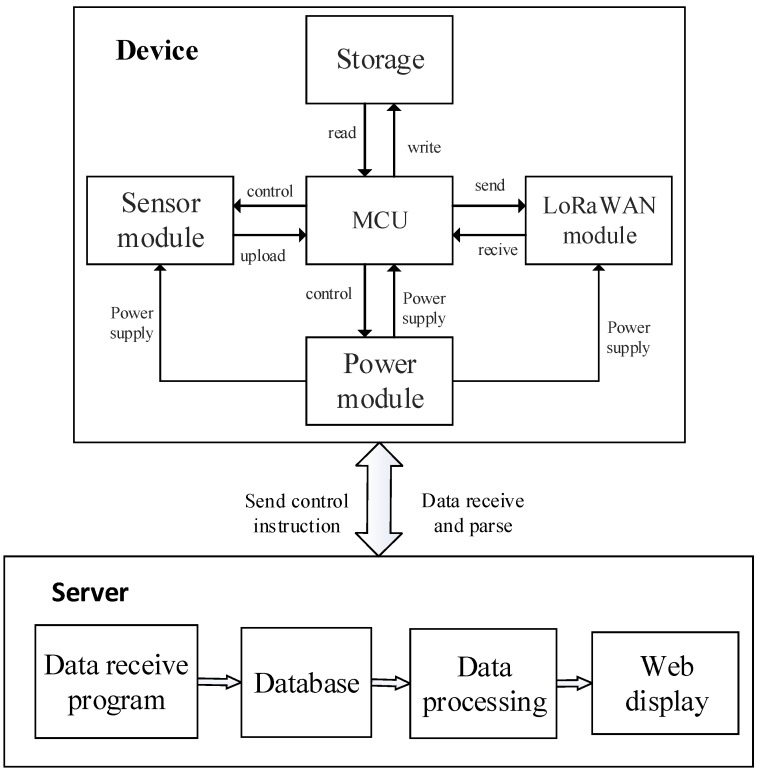
LoRa monitoring system consists of monitoring devices (**top**) and a data server (**bottom**). Arrows between different modules shown data flow and module control procedures. MCU = microcontroller unit; WAN = wireless area network.

When monitoring the air quality, it is essential to use a high-density device layout because the environment is usually both complex and volatile [14]. First, the devices must remain sufficiently stable to ensure a long-term monitoring capability. In general, because of the limited access to a mains power supplies in most scenes, the devices are required to be powered by solar energy [15] and they must meet low power consumption standards. Second, it should also be a simple process to replace sensors in the proposed devices to allow the system to collect different types of pollutants. The monitoring equipment that is described in this article is designed in modular form [16], which means that each module’s functionality will be realized in accordance with specific application requirements. To meet the requirements for low power consumption and on-line collection of atmospheric pollutants and PM concentrations, the system is largely designed in two parts, i.e., the device side and the server side (as shown in Figure 2). The device consists of the following components: a central control processor module, a sensor data collection module, a data storage module, a power module, and the LoRa communication module. Each module is connected to the processor and performs data collection, storage, and remote transmission tasks via coordination of the software.

### 2.1. Node Device

The central control processor is mainly based on the STM32F107 [17] chip that is manufactured by STMicroelectronics at Geneva, Switzerland, which is based on a chip from the 32 advanced RISC (Reduced Instruction Set Computer) machine (ARM) Cortex™-M3 series, with a rich array of peripherals and sufficient random-access memory (RAM) space. This module controls the functions of the entire system, including data processing, data storage, and the software drivers required for the communication module and power system control.

The sensor data collection module largely contains electrochemical gas sensors (for SO_2_, CO, O_3_, and NO_2_) and laser scattering sensors (PM2.5 and PM10 particulates). Their specific parameters are shown in Table 1. The sensor interface uses a serial port mode. To ensure that sufficient numbers of serial port sensors can be connected, the system uses a digital switch to poll the sensor data, thus effectively reducing the required number of microcontroller unit (MCU) serial ports.

The data storage module uses a serial peripheral interface flash memory (SPIFLASH) [18] to store the relevant data. There may be some scenarios in which the communication connection is disconnected for a period of time. Therefore, to ensure the continuity of the collected data, it is necessary to store any data that cannot be uploaded in time. At the same time, the system’s power consumption can be further reduced by reducing the frequency with which data is uploaded.

The LoRa module controls the wireless data transmission functions of the device. The module is connected to the base station using the LoRaWAN protocol [19]. During periods where there are no data uploads, the module operates in a very low power consumption state. However, when there is an upload, the device is woken up to perform the data upload and receive the downlink data from the base station.

The power module controls the power supply to the hardware system. The power supply uses a combination of solar energy and a battery to ensure that the device can work normally in remote locations where there is no access to a mains supply. In accordance with the formulated power supply strategy, the module will supply power to other modules when required, and the power supplies of these other modules will be turned off if there is no task to be performed, which will save energy to a great extent.

### 2.2. Server Side

The server side is in charge of receiving, passing, storing, and displaying the node device data. The data reception program is based on the Socket communication real-time online monitoring network port. When data are received by the program, these data are parsed and are then written into the MySQL database using the database storage table field. The webpage will then query the database periodically. When there is a new data update, this update will be analyzed with reference to the historical data, and the results will be displayed on the webpage, thus fulfilling the visualization and readability requirements for the monitoring data.

## 3. System Design

The system was designed using a combination of software and hardware. The software includes a software platform based on the browser/server (B/S) [20] framework and the embedded system software. The hardware is the front-end data acquisition main board. To meet the demand for collection of atmospheric pollutant concentrations, the system uploads the collected data using the LoRaWAN protocol. The system platform design is divided into hardware, software, and system key technology design aspects, and each of these aspects will be introduced in detail below.

### 3.1. Hardware Design

#### 3.1.1. Main Board

**Figure 3:**
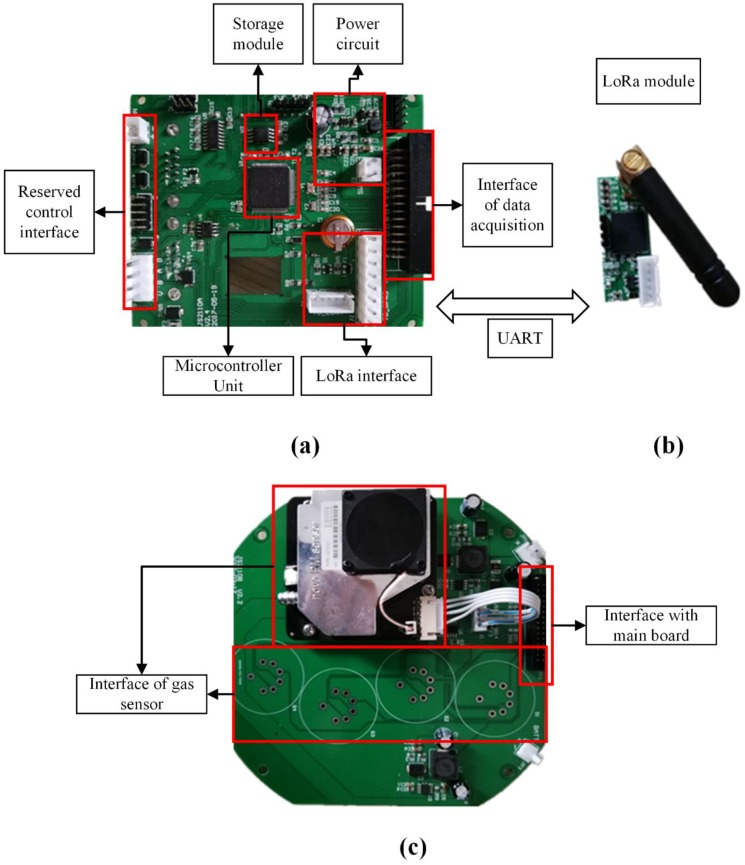
LoRa acquisition equipment hardware structure, consisting of three parts. (**a**) Main board, (**b**) LoRa module, (**c**) Data Collection Board. UART = universal asynchronous receiver/transmitter.

The main board is pictured in Figure 3. As the core component of the atmospheric monitoring node, the main board is composed of a microcontroller, a peripheral circuit, a charge management unit, a power conversion unit, a J-Link component, a serial port setting unit, a data storage unit, a clock management unit, a network interface, and a signal transceiver. Using an STM32F107 chip and the embedded Beijing Normal University Operation System (BNUOS) which developed by the State Key Laboratory of Remote Sensing Science of Beijing Normal University, the microprocessor controls data acquisition in the field environment and data storage, and also manages communication between the collector and the remote server, and communication between the collectors.

#### 3.1.2. Data Collection Board

Most scientific research requires simultaneous observation of multi-parameter data, which means that a single set of equipment must be used to connect multiple different types of sensors at the same time or to connect equipment from different manufacturers (or even from different countries). To improve the versatility and extensibility of these platforms and support flexible collocation of the different components and devices in a diverse range of projects, it is necessary to design interface modules with increased numbers of functions. Therefore, the main board and the data collection board are separated on these platforms. While the main board is integrated with the serial port, Universal Serial Bus (USB), and Ethernet ports that are common to most current sensors, the data collection board is designed separately to connect each of the sensors. For digital sensors, the data collection board provides a sensor-to-processor communication interface; for analog sensors, if the sensor signal is to be amplified and converted, the data collection board provides the functional components required to link the final signal to the processor; for the output pulse sensor, the data collection board shapes the pulse that is output by the sensor and connects this pulse to the processor. In addition, the data collection board is also both scalable and customizable. The input/output (IO) port on the main board, which is connected to the data collection board, contains 12 analog-to-digital converters (ADCs) and 12 digital-to-analog converters (DACs), a universal synchronous and asynchronous receiver-transmitter (USART), a controller area network (CAN), an inter-integrated circuit (I^2^C) bus, a timer, and a counter. In addition to the analog and digital signals, sensors that output other signals such as universal synchronous and asynchronous receiver (USAR), recommended standard 485 (RS485), CAN, I^2^C, and serial digital interface at 1200 baud (SDI12) sensors can be connected to the data collection board. Therefore, these sensors can be customized depending on specific demands.

On the platform, the data collection board is as shown in Figure 3; through this board, the sensors can communicate and provide data interaction with the main board. For the different types of sensors described above, the data collection board interacts with the main board in different ways. For example, for the digital sensors, the data collection board only provides an interface to allow these sensors to communicate with the main board. For the analog sensors, however, the data collection board amplifies and converts the electrical signals that are output by the sensors, and then hands them over to the main board via special interfaces for processing.

### 3.2. Software Design

#### 3.2.1. Software Design of Server

When the central server processes the data, a source analysis model and the weather element analysis environmental quality trends are established in combination with the actual data. The software platform is thus designed based on the demands described above.

The atmospheric environmental monitoring system software is created in the Django framework [21], which stores data using a MySQL database and communicates with the terminal collection device via Socket communication. The terminal application software that receives the device uploads the coded data via a data network, and then analyzes, stores, and displays the data while also providing data visualization and downloading functions for further data analysis. The application software has two main functions: data reception and storage, and real-time data analysis and publication.

**Figure 4:**
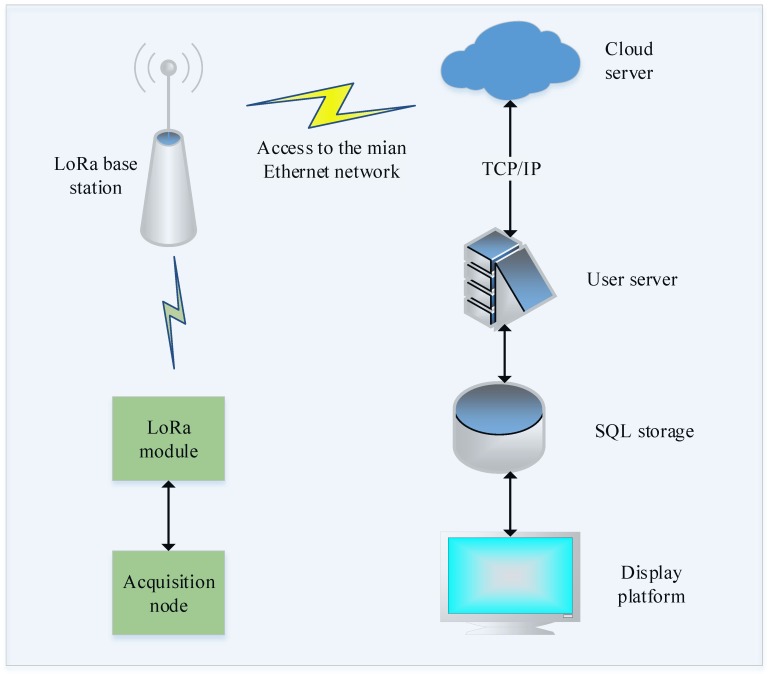
The data communication connection between the user server and the device. TCP/IP = transmission control protocol/internet protocol; SQL = structured query language.

The data communication connection between the user server and the device is illustrated in Figure 4. The device is connected to the LoRa base station through the star topology of the LoRaWAN protocol. The LoRa base station accesses the backbone network using the general packet radio service (GPRS), Wi-Fi [22], or Ethernet connections, and completes the network connection to the cloud server through the TCP/IP (Transmission Control Protocol/Internet Protocol) Internet protocol suite [23]. The user server transmits the data to the cloud server via stream sockets, which provide connection-oriented, two-way, and ordered data flow services based on the TCP. The user server application software collects the data that are transmitted by the terminal device via the Socket data receiving stream to the server data pool. The application program then obtains the corresponding data bits based on the data sequence that was transmitted by the device and the corresponding sensor flag bits, and thus acquires the real data using the corresponding conversion factors after the data bits are translated into collection data. The server application program then stores the data in the MySQL relational database. After the receiving program receives the data that are sent by the terminal device and decodes these data, it converts the data into the corresponding data model required for the data call.

The application program visualizes the collected data in accordance with the demands of the users. The overall data visualization framework used here is the popular web framework, Django. The corresponding request link is sent to the background server via the address request of the foreground and the server then obtains the corresponding processing function via the corresponding route mapping. The processing function performs the data processing required, and then the data obtained after processing are fed back to the foreground page for display rendering. Depending on the users’ demands, the data analysis device can conduct hourly average, daily average, monthly average, and air quality index (AQI) [24] calculations of these data and the corresponding analysis results are then fed back to the users.

In the system, according to the LoRa base station manufacturer introduction and application results, 1000 collection nodes can be supported under one LoRa base station [25]. The communication rate of LoRa is 292 bps–5.4 kbps, so communication delay is very short. The system has six digital sensors: O_3_, SO_2_, NO_2_, CO, PM2.5, and PM10. In practical application, considering the sensor startup time is <30 s (as shown in Table 1), the acquisition frequency can approach 1 min, and the acquisition frequency can be set by sending control data to the device remotely, so the collection of the main basic parameters of the atmosphere can be completed. The data requirements for environmental governance analysis can be met through these six parameters.

#### 3.2.2. Embedded Software Design

(1)   Main System

**Figure 5:**
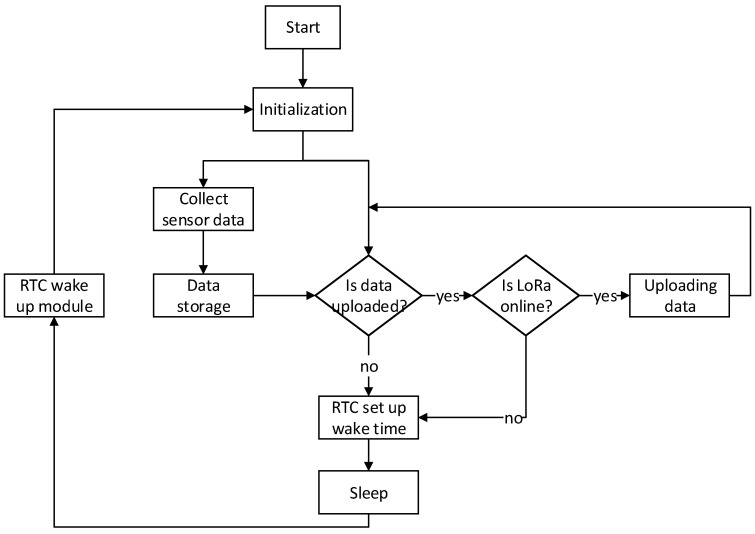
The embedded platform software flow that is applied to the main board of LoRa acquisition equipment. It shows the processing of an acquisition task. RTC = real time clock.

The embedded platform software flow is depicted in Figure 5. To reduce the power consumption of the system, the main task module has a system sleep function. Before the system can enter the sleep state, it must determine the tasks that are to be executed after the next system wake-up. The main tasks are as follows: 1. performing the sampling task; 2. opening the LoRa communication module and uploading the sampled data; and 3. opening the LoRa communication module and testing whether or not the link with the server is normal.

In this system, there are several places where the sleep program can be called or the sleep flag can be set. However, the sleep state is only allowed after the sampling and data transmission tasks have been completed or if the battery voltage is below the minimum level required.

In addition, in the case where there is a setup tool or a setup software connection, the system will also wake up automatically.

(2)   Timing Module

The timing module has two different situations. 1. In systems with a sleep function, the timing module provides a trigger to start the sampling process. In these systems, the turn-on times of the data transmission module and the timing module are preferably staggered. 2. In systems that work continuously without a sleep function, the timing module provides semaphores to start the sampling and data transmission modules. In addition, sampling, data transmission, and Global Positioning System (GPS) time calibration should also be controlled via setup tools or using remote networks.

(3)   Wireless Transmission

The wireless data transmission module should be formed using standard send-receive functions. The main tasks of this module are listed as follows: 1. Uploading the data collection information and storing this information in an external flash memory; 2. Uploading the signal intensity and the real-time battery voltage of the module while simultaneously uploading the data to enable observation of the current state of the system; and 3. Setting the system parameters and uploading the system status information via remote control. All required correspondences are checked and confirmed.

(4)    Data Storage

In general, the system data storage is divided into two parts: 1. the sample data, which are stored in pieces and 2. the system work log information, which includes the information and the status when the system is working, including the times at which the communication module starts and the reasons why the system goes into sleep mode.

## 4. Key Technology

### 4.1. System Operational Stability

Given that these devices commonly operate under the condition that there is no on-site maintenance, self-healing capabilities are strongly required for the device. During the early test procedures, the device halted several times in the unadjusted state and only returned to normal when the device was restarted manually. Usually, these problems are caused by abnormalities in the hardware and software systems. In a mono-chip microcomputer system, data confusion among the various registers and the memory will cause a program pointer error, the pointer not in program area error, and the wrong program instructions, which will interrupt normal operation of the program. As a result, the system that is controlled by this mono-chip microcomputer cannot work normally, meaning that it will stagnate and then finally halt. Therefore, when the system was designed, a hardware watchdog circuit and a software timing reset were installed to ensure that the system can recover spontaneously when any such abnormality occurs. The watchdog is a circuit that checks the internal workings of the chip periodically and issues a hardware reset signal to the chip in the event of an error.

**Figure 6:**
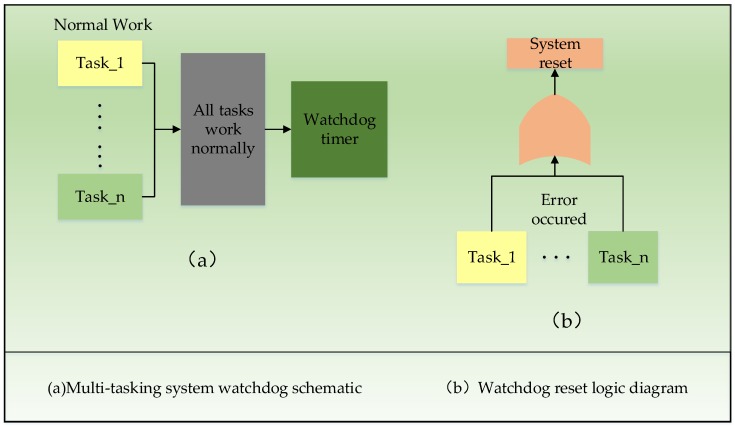
The operational process of the watchdog within the system, which is applied to the main board of the LoRa acquisition equipment in order to ensure the device works normally.

The operational process of the watchdog within the system is as shown in Figure 6. The system contains multiple tasks in the group of Task 1, Task 2 … Task *n*, and a task monitor which is superior to other monitored tasks. Under the condition that Task 1 to Task *n* all work normally, the Task Monitor clears the hardware watchdog timer within a specified period of time. In the case where any task, e.g., Task *x*, breaks down, the Task Monitor then does not clear the hardware watchdog timer so that the system will reset automatically when the monitored task fails. Additionally, if the Task Monitor itself breaks down, it obviously cannot clear the timer in time, and the watchdog can also reset automatically in that scenario.

### 4.2. LoRa Communication Design

**Figure 7:**
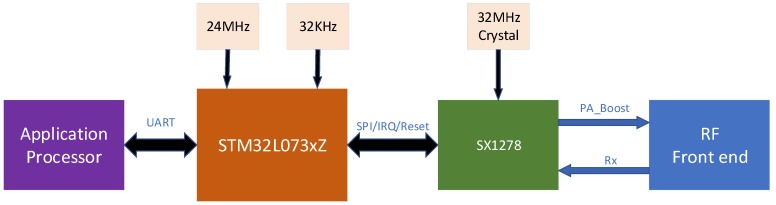
The design hardware structure of LoRa module. SPI = serial peripheral interface; IRQ = interrupt request; PA = power amplifier; Rx = receive; RF = radio frequency.

The system uses the LoRaWAN protocol chip SX1278 that is manufactured by Semtech [26], which provides long-distance spread spectrum communication based on the standard LoRa modulation technique. The frequency bandwidth of the chip is between 7.8 kHz and 500 kHz. The SF (Spreading Factor) range is from 6 to 12. The available frequency range is from 137 to 525 MHz. To simplify the use of each module in the proposed system, a low-power-consumption chip manufactured by STMicroelectronics, STM32L073xZ, is used specifically as the drive-control chip for the SX1278, with the design structure shown in Figure 7. In this design scheme, the LoRa communication is encapsulated in a transparent transmission module, and the upper application processor transmits and receives the data through the serial port; this not only simplifies the design structure, but also allows the LoRa module to be used in other applications more easily.

**Figure 8:**
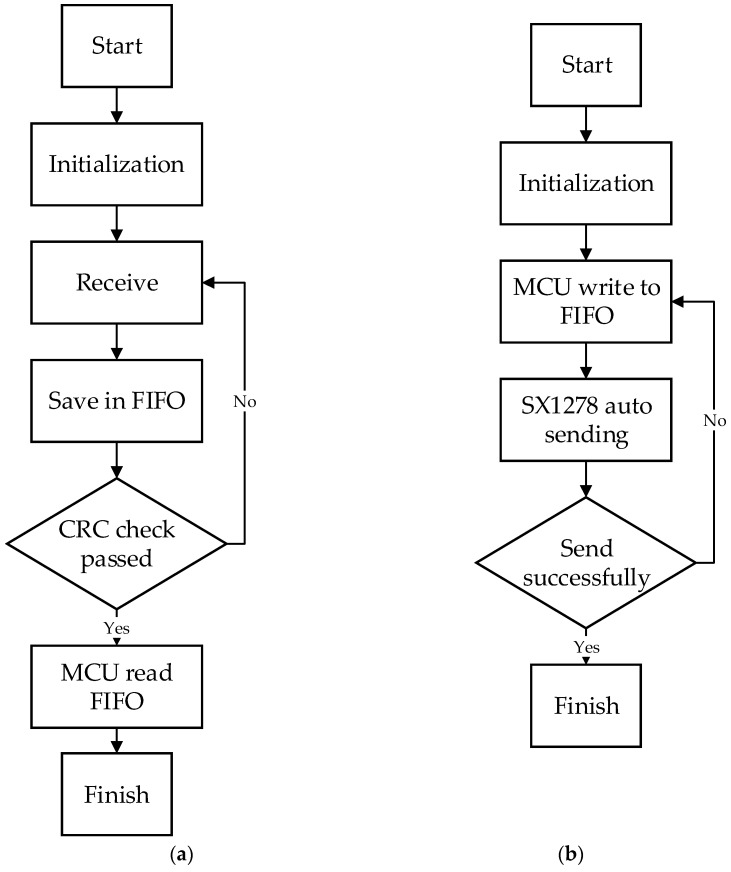
LoRa communication processing between the LoRaWAN chip SX1278 and the MCU of the main board. (**a**) LoRa receiving data. (**b**) LoRa sending data. CRC = cyclic redundancy check; FIFO = first input first output.

The communication process of SX1278 follows the LoRaWAN communication protocol [19]. The LoRaWAN network architecture is a star topology. There are three work modes: Class A, Class B, and Class C, and Class C is used in the system. When LoRaWAN is transmitting, CRC (cyclic redundancy check) is used to ensure sending data correctness. Each LoRa terminal node has its own unique media access control (MAC) address. When the network environment is configured successfully, the sending data includes MAC, channel, and payload. LoRa has a 256 byte data first input first output (FIFO) buffer, which stores the data that is received or will be sent. MCU reads and writes data by accessing FIFO. The receiving and sending flow is shown in Figure 8. In the receiving state, the LoRa module saves the received data in FIFO and carries out CRC checking. After checking successfully, MCU will read the data from FIFO then finish the receiving process. In the sending state, the MCU writes the data into FIFO, and SX1278 automatically sends the data. When the sending is successful, the MCU receives the response message, then the transmission is completed, otherwise the data will be re-transmitted.

## 5. Application Analysis

To test the device’s stability and the accuracy of the collected data, monitoring devices were actually installed at multiple points and operated for long periods to acquire the data. The historical operational behavior of the data analysis devices and the final conclusions are presented as follows.

### 5.1. Application Stability

The data as shown in Table 2 were collected using several station devices over a period of three months from January to April in 2017 under zero maintenance conditions. The table shows that the data integrity rate of the device is generally above 90%, which indicates that the device did not shut down during this time period. The main reasons for loss of any data are as follows. First, the LoRa communications may be mis-coded or lost during upload, because the distance between the base station and the device will affect LoRa communications. If the device is more than 2 km away from the base station, the data loss rate will then be greatly increased. Second, the device may be abnormal as a result of the effects of the external environment. The watchdog and timing reset strategies are thus applied to ensure that the device can recover spontaneously. It was demonstrated that the system meets the requirements for use in long-term atmospheric environment monitoring.

### 5.2. Data Validity

**Figure 9:**
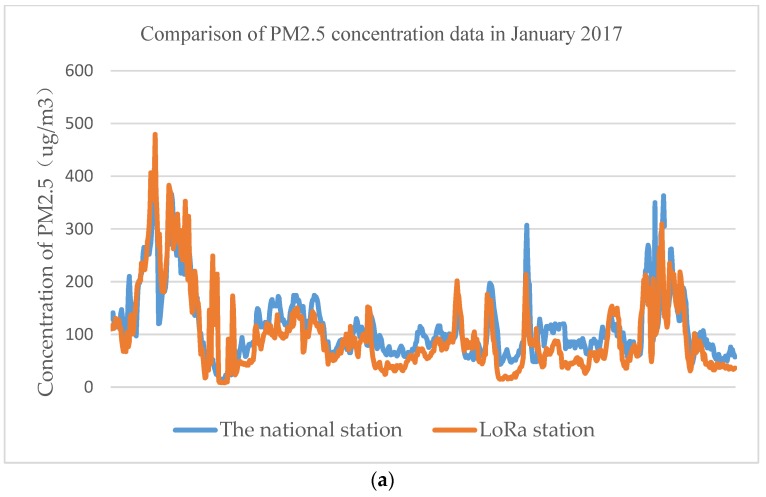

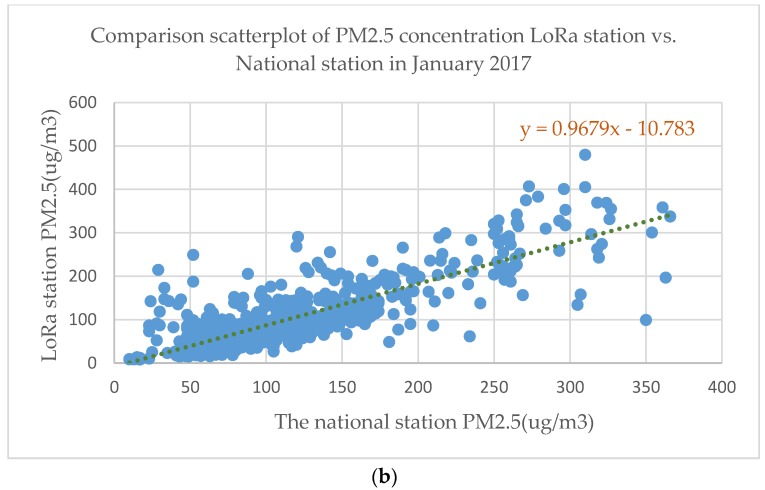
Comparison between the authoritative data that were issued by the national station and the LoRa device station for the concentration of PM2.5 in a month. (**a**) Comparison line graph of the national station data and the LoRa PM2.5 data in a month. (**b**) Comparison scatter plot of the national station PM2.5 data and the LoRa PM2.5 data in month.

**Figure 10:**
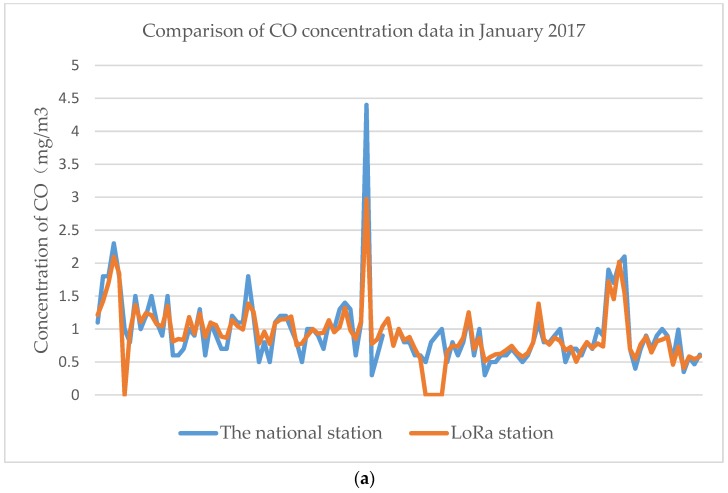

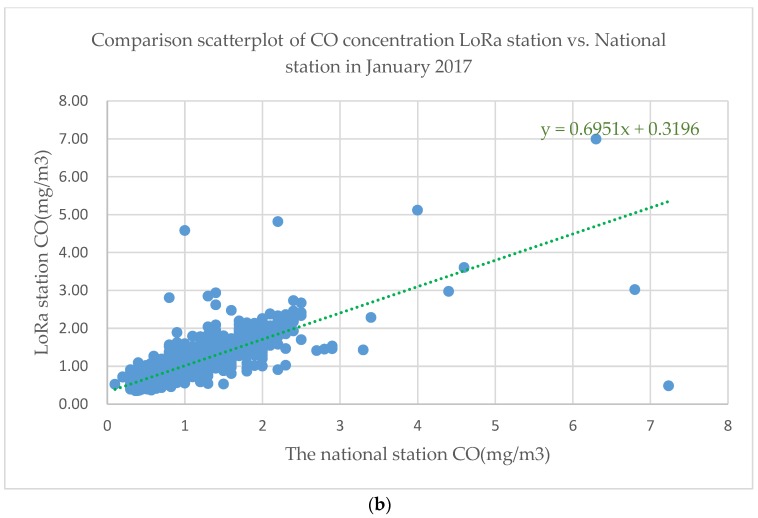
Comparison between the authoritative data that were issued by the national control station and the LoRa device station for the concentration of CO in a month. (**a**) Comparison line graph of the national station data and the LoRa CO data in a month. (**b**) Comparison scatter plot of the national station CO data and the LoRa CO data in month.

To check the validity of the data that were collected using the device, the data were compared with the authoritative data that were issued by the national control station for the concentration of PM2.5 and CO, which has been causing concern recently, for the whole month. The change trends (as shown in Figure 9 and Figure 10) in the data that were collected using the proposed device based on the LoRaWAN protocol that we introduced here are basically the same as those of the national control station, thus, fully demonstrating that the monitoring and tracking of pollutants can be performed using the proposed system. Practical application tests have shown that the device has full practical application capabilities; its monitoring points can be disposed with high density to meet the application requirements for pollution emission source location under the condition where a large part of the cost must be limited.

### 5.3. Time Delay

The time delay of the proposed platform system mainly consists of data collection delays and LoRa communication delays. The data collection delay is caused by the start-up times of the sensors. In this system, PM2.5 and PM10 particulates are monitored using laser light scattering sensors [27], which have a start time of less than 10 s. However, the remaining SO_2_, NO_2_, and other gas pollutants are monitored using electrochemical sensors, which require more than 30 s for startup configuration under the manufacturer’s instructions. Additionally, the average time taken by the device to collect the data from the sensors is approximately 32 s. LoRa communication delays are mainly caused by the time taken for LoRa to be registered in the network. Practical applications show that communication delay is less than 5 s when the network signals are strong, while the registration time may be up to 25 s when the signals are weaker. Under the worst-case conditions, the time delay may be as much as 57 s.

### 5.4. Precision

The system sensors are all digital. Because the sensors that monitor the PM2.5 and PM10 particulates are all designed based on the laser scattering principle, the measurement precision may be affected by foggy weather, during which the tested air would need to be dried for accuracy. The sensors for SO_2_, NO_2_, O_3_, and CO are all based on the electrochemical principle [28], which means that both the temperature and the air pressure easily affect their measurement precision. Therefore, in practice, the data will need to be compensated and calibrated.

### 5.5. Power Consumption

**Figure 11:**
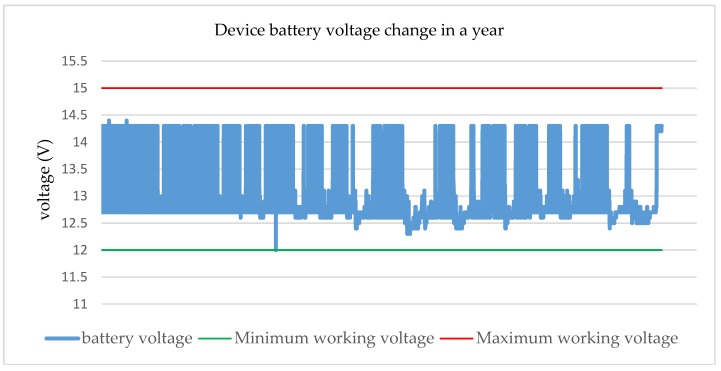
The battery voltage change trend of the device in a year. It shows that the battery voltage (the blue line) was always maintained between the maximum (the red line) and the minimum working voltage (the green line), and the solar-based power supply ensured that the equipment was working properly.

The system power supply uses a combination of solar energy and battery power. In the practical tests, for operation with low power consumption, the power supply can completely meet the monitoring system requirements. As shown in Figure 11, the battery voltage fluctuation of the device was basically maintained within the 12 V–14.5 V range for the entire year, thus indicating that the device has not experienced insufficient power supply conditions.

## 6. Conclusions

Using LoRa communication, the proposed system has performed real-time monitoring of atmospheric pollutant concentrations and the hardware and software design of the platform has been completed. The LoRa module can reduce both the power consumption and the cost of the device efficiently. Furthermore, the combination of the ARM processor with its low power consumption together with the system having a sleep function allows the device to collect data at high frequency for long periods, powered only by solar energy and a battery. The long-term practical tests proved not only the stability and the reliability of the proposed device, but also showed that some of the data may be lost when the distance between the LoRa base station and the nodes is too great or when there are too many obstructions between the base station and the nodes. Therefore, in future work, we will continue to improve the software system to enable the detection and retransmission of lost data, to allow remote upgrading of the device through the server, and to enable adjustment of the sensor parameters. Furthermore, we intend to continue to develop sensors that are compatible with other environmental parameters, such as soil and water parameters, based on the foundation of the system proposed here. Finally, we hope to achieve intelligent environmental monitoring using the advantages of LoRa in the IoT to provide a reliable and stable data reference source for monitoring and management of environmental problems.

## Figures and Tables

**Table 1:** The atmospheric sensors’ specific parameter characteristics (model, measuring range, measuring principle, resolution, response time, accuracy) used in the system. PM = particulate matter.

Type	Model	Measuring Range	Measuring Principle	Resolution	Response Time (s)	Accuracy
O_3_	7NE/O3-5	0–2000 μg/m^3^	Electrochemistry	2.0 μg/m^3^	<30	≤±5%
SO_2_	7NE/SO2-1000	0–2500 μg/m^3^	Electrochemistry	2.5 μg/m^3^	<30	≤±5%
NO_2_	7NE/NO2-1000	0–2000 μg/m^3^	Electrochemistry	2.0 μg/m^3^	<30	≤±5%
CO	7NE/CO2-1000	0–200 mg/m^3^	Electrochemistry	0.2 mg/m^3^	<30	≤±5%
PM2.5	SDS011	0–2000 μg/m^3^	Laser scattering	0.1 μg/m^3^	<10	≤±10%
PM10	SDS012	0–2000 μg/m^3^	Laser scattering	0.1 μg/m^3^	<10	≤±10%

**Table 2:** Actual acquisition comparison of multiple station. Theoretical data size (TDS) indicates the volume of data that should be acquired at the normal acquisition frequency within three months. Actual data sizes (ADS) indicates results that are counted by real data. Then, using the equation data acquisition efficiency DAE = ADV/TDV acquired the data integrity rate of the device.

Station	TDS	ADS	DAE
Station-1	2712	2712	100%
Station-2	2712	2559	94.36%
Station-3	2712	2563	94.51%
Station-4	2712	2694	99.34%
Station-5	2712	2692	99.26%
Station-6	2712	2404	88.57%
Station-7	2712	2596	95.72%
